# Intranasal natural products for influenza treatment: a systematic review and meta-analysis of preclinical studies

**DOI:** 10.3389/fimmu.2026.1758831

**Published:** 2026-03-10

**Authors:** Kaiqi Zhang, Yao Lu, Jingwen Yang, Changqi Shi, Ning Zhao, Xiaojuan He, Cheng Lu, Li Li

**Affiliations:** Institute of Basic Research in Clinical Medicine, China Academy of Chinese Medical Sciences, Beijing, China

**Keywords:** influenza, intranasal, meta-analysis, natural products, systematic review

## Abstract

**Background:**

Influenza imposes a heavy global public health burden, with current therapies limited by drug resistance and side effects. Natural products have antiviral potential, and intranasal delivery targets the respiratory tract. However, preclinical evidence lacks systematic evaluation, necessitating this review.

**Methods:**

This study is a systematic review and meta-analysis conducted per the PRISMA guidelines. Studies meeting the inclusion criteria were retrieved and screened from the PubMed, Embase, Web of Science, China National Knowledge Internet, VIP Information Chinese Periodical Service Platform, China Biology Medicine Disc, and Wanfang Data Knowledge Service Platform databases. A meta-analysis was performed using R Studio software. The mean difference (MD) and relative risk (RR) were calculated using fixed effects or random effects models. Sources of heterogeneity, sensitivity, and publication bias were also explored.

**Results:**

A total of 23 studies were included. The results of the meta-analysis revealed that compared with the control treatment, intranasal natural products significantly increased the survival rate (RR = 3.47, 95% confidence interval [CI]: 2.44 to 4.93, p < 0.001) and reduced the viral titer (MD = −1.30, 95% CI: −2.27–−0.32, p=0.0092 < 0.01) and lung index (MD =−0.10, 95% CI: −0.19–−0.02, p = 0.015 < 0.05). In addition, intranasal natural products exerted regulatory effects on the body weight and inflammatory cytokines of influenza-infected mice.

**Conclusion:**

The results show that intranasal natural products significantly increase survival and reduce the lung viral load in influenza models, with preliminary exploration of the underlying mechanisms and therapeutic potential. Owing to methodological limitations and heterogeneity, high-quality preclinical studies and standardized animal experiments are needed.

**Systematic review registration:**

https://inplasy.com/, identifier INPLASY INPLASY2025120006.

## 1 Introduction

Influenza virus infections are infectious viral diseases characterized by high morbidity and mortality; these infectious diseases can trigger global pandemics (1). Clinical manifestations vary in severity: mild cases present only as common colds, with short disease duration, mild symptoms, and favorable prognosis, whereas severe cases may progress to influenza viral pneumonia or even be life-threatening (2).

At present, antiviral drugs are the mainstay for influenza treatment, with oral and intravenous administration as the mainstream routes. In recent years, intranasal administration, which can act directly on target sites in the respiratory tract and reduce systemic side effects, has gradually emerged. Zanamivir, which is recommended by clinical guidelines (3), recombinant human interferon α-1b, which is used in clinical practice (4), ribavirin (5), and inhaled vaccines for prevention (6), have all adopted inhalation administration and been applied in clinical practice or prevention scenarios, further confirming the value of this administration trend. The advantages of intranasal administration are closely associated with its unique physiological structures: The nasal mucosa, as part of the respiratory epithelial tissue, allows intranasal administration to exert local effects through direct drug delivery and systemic effects via capillary absorption in the submucosa (7). Furthermore, the lungs possess a more abundant physiological structural basis: The lungs, with 300 million alveoli and more than 280 billion capillaries for gas exchange, have a blood flow of 5,700 mL/min. This high vascularization enables rapid drug absorption via intranasal administration to induce systemic effects (8).

Currently, the variety of available drugs in the field of intranasal administration remains relatively limited. Natural products have a wide range of sources, including plants, animals, and minerals, providing abundant resources for the screening of active ingredients suitable for intranasal administration and enabling the exploration of candidate drugs targeting the pathological mechanisms of respiratory diseases. Among them, natural products such as Scutellaria baicalensis, Angelica dahurica, and Peganum harmala have been confirmed to possess anti-influenza virus activity (9), which can inhibit viral infection and delay disease progression (10). After local administration of natural products via the respiratory tract, the drugs can act directly on target sites of the respiratory mucosa, and the dose of drugs entering the systemic circulation is extremely low, which can significantly reduce the risk of systemic exposure (11). Therefore, the toxicity and side effects of natural products are relatively mild. This characteristic is highly consistent with the high safety requirements for local respiratory mucosal administration and can minimize the incidence of adverse reactions.

Systematic reviews and meta-analyses of preclinical studies enhance the reliability of results, facilitate clinical translation, and underscore their pivotal significance (12). Specifically, such analyses have been conducted for preclinical studies on natural products in fields such as health span improvement and premature ovarian failure (13, 14). Therefore, a systematic review and meta-analysis of preclinical studies on natural products for influenza treatment via intranasal administration are needed.

## 2 Method

The review was conducted in accordance with the preferred reporting items for systematic reviews and meta-analyses (PRISMA) guidelines (15). The study was formally registered on INPLASY (https://inplasy.com/; registration number: INPLASY2025120006).

### 2.1 Search strategies

Two authors (KZ and JY) independently conducted searches of seven electronic databases—PubMed, Embase, Web of Science, China National Knowledge Internet (CNKI), VIP Information Chinese Periodical Service Platform (VIP), China Biology Medicine Disc (CBM), and Wanfang Data Knowledge Service Platform (Wanfang)—to identify relevant animal studies from database inception until December 2025. We used medical subject headings (MeSH) and free-text terms in our search, which were tailored for each database without language or publication year restrictions. The MeSH terms were as follows: (“influenza, human” OR “influenza A virus” OR “influenza B virus” OR “orthomyxoviridae”) AND (“biological products” OR “phytochemicals” OR “drugs, Chinese herbal” OR “herbal medicine” OR “phytotherapy” OR “plant extracts” OR “medicine, traditional” OR “medicine, east Asian traditional” OR “medicine, Korean traditional” OR “medicine, Tibetan traditional” OR “medicine, Mongolian traditional” OR “medicine, African traditional” OR “medicine, Chinese traditional” OR “medicine, Iranian traditional” OR “oils, volatile” OR “perfume”) AND (“administration, intranasal” OR “nasal absorption” OR “nebulizers and vaporizers” OR “administration, inhalation” OR “Nasal sprays” OR “aromatherapy”) AND (“animals” OR “animal experimentation”).

The free-text terms were as follows: (“influenza” OR “flu”) AND (“biologic* product*” OR “naturally product*” OR “biopharmaceutical*” OR “biologic*” OR “plant derived compound” OR “plant bioactive compound” OR “plant derived chemical” OR “phytochemical” OR “phytonutrient” OR “pharmacognosy” OR “ethnobotany” OR “ethnopharmacology” OR “traditional medicine” OR “medicine east Asian traditional” OR “medicine Korean traditional” OR “medicine African traditional” OR “Traditional Chinese Medicine” OR “medicine Iranian traditional” OR “perfume” OR “volatile oil” OR “essential oil”) AND (“intranasal administration” OR “intranasal drug administration” OR “nasal administration” OR “atomizer*” OR “inhaler*” OR “nebulizer*” OR “vaporizer*” OR “Inhalation” OR “aerosol” OR “respiratory drug administration” OR “nasal spray” OR “nasal mist” OR “intranasal instillation” OR “aroma*”) AND (“animals” OR “animal experimentation” OR “rat” OR “rats” OR “mouse” OR “mice” OR “swine” OR “porcine” OR “murine” OR “sheep” OR “lambs” OR “pigs” OR “piglets” OR “rabbit” OR “rabbits” OR “cat” OR “cats” OR “dog” OR “dogs” OR “cattle” OR “bovine” OR “monkey” OR “monkeys” OR “trout” OR “marmoset” OR “bird*” OR “avian”). **Supplementary Table 1** provides details about the search strategy.

### 2.2 Inclusion and exclusion criteria

The inclusion criteria were based on the PICO principle: (1) Participants: Animal models infected with influenza virus; (2) Intervention: Influenza intervention using natural products via intranasal administration (e.g., nebulized inhalation, intranasal instillation, aerosol inhalation, etc.); (3) Comparison: The control group was an influenza virus-infected model group without treatment; (4) Outcomes: Primary outcomes included survival rate; secondary outcomes included viral titer, lung index (calculated as: Lung index = (Lung wet weight/Body weight) × 100%), inflammatory cytokines, and body weight. Studies that included at least one of the above five outcomes were eligible for inclusion.

The exclusion criteria were as follows: (1) duplicated published literature; (2) reviews and editorials; (3) *in vitro* studies, clinical trials, or computer-based studies; (4) studies focusing on influenza prevention; (5) studies using nonintranasal administration routes; and (6) studies involving nonviral infections (e.g., bacterial or fungal infections).

### 2.3 Data extraction

The retrieved literature was managed using NoteExpress (version 3.7). After deduplication, two authors (KZ and JY) independently screened the studies in accordance with the predefined inclusion and exclusion criteria. Any discrepancies arising during the screening process were resolved through the corresponding author (LL). Essential information from the eligible studies was extracted and recorded using Excel 2019 software, covering the following dimensions:

1. Publication details: First author and publication year;
2. Animal characteristics: Species, strain, age, and weight of the influenza virus-infected model animals;
3. Modeling method: Specific protocol for establishing the influenza virus-infected animal model;
4. Sample size: Number of animals in the experimental group (T) and the model control group (M);
5. Intervention details: Nature of the natural products, administration route, intervention dose, and duration of administration;
6. Observation duration: Time span of outcome monitoring starting from influenza virus infection;
7. Outcome measures: survival rate, viral titer, lung index, inflammatory cytokines, and body weight.

For multiple studies containing similar data, only the one published earliest or with the largest sample size was selected. If a study evaluated multiple natural products, each treatment was analyzed separately as distinct original research. When a study conducted measurements at multiple time points or with multiple doses, all the measured values were extracted; however, only the data corresponding to the lowest effective dose and the last time point were included in the final meta-analysis. When outcome data were presented in graphical format only, attempts were made to contact the corresponding authors of the relevant studies to obtain raw numerical data. If raw data could not be acquired, the graphical results were quantified using Origin 2024 to extract mean values and other necessary statistics (16). For all studies with graphically presented data, the digitization process was conducted strictly in accordance with standardized protocols: two researchers independently performed the data extraction, and a third independent researcher was consulted to mediate and resolve any discrepancies arising during this process.

### 2.4 Bias assessment

The quality of the included studies was independently evaluated by two investigators (KZ and JY) using the SYRCLE risk-of-bias assessment tool for animal experiments. The evaluation criteria were as follows: (1) random sequence generation; (2) baseline characteristics; (3) allocation concealment; (4) random animal housing; (5) blinding of researchers; (6) random outcome assessment; (7) blinding of outcome assessment; (8) incomplete outcome data; (9) selective outcome reporting; and (10) other types of bias. “Yes” indicated low risk, “no” indicated high risk, and “unclear” indicated insufficient information to correctly assess the risk of bias (17). Any disagreements that arose during this phase of the project were resolved through the corresponding author (LL).

### 2.5 Statistical analysis

Data from the included studies were statistically analyzed using R Studio software. Heterogeneity among studies was assessed via the I² test. If the heterogeneity test results were P ≥ 0.1 and I² < 50%, indicating low heterogeneity between studies, a fixed-effects model was used for pooled effect size analysis; if P < 0.1 and I² ≥ 50%, indicating high heterogeneity between studies, a random-effects model was adopted for pooling. The effect sizes of the outcome measures were reported as follows: dichotomous outcomes were quantified using RR with 95% CI, and continuous outcomes were described using MD with 95% CI. Statistical significance was determined by P < 0.05, and for dichotomous outcomes, the 95% CI did not include 1. The statistical heterogeneity of effect sizes was further quantified and evaluated using the I² value. We conducted a sensitivity analysis for the primary outcome measure (survival rate) in our meta-analysis by systematically excluding each study. This allowed us to assess the impact of the excluded study on the overall results and identify any influential studies. We used a funnel plot and Egger’s test to assess the publication bias of the primary outcome measure (survival rate). In case of publication bias, we applied the trim-and-fill method to adjust for this bias by identifying and supplementing theoretically missing studies.

## 3 Results

### 3.1 Results of literature screening

**Figure 1:**
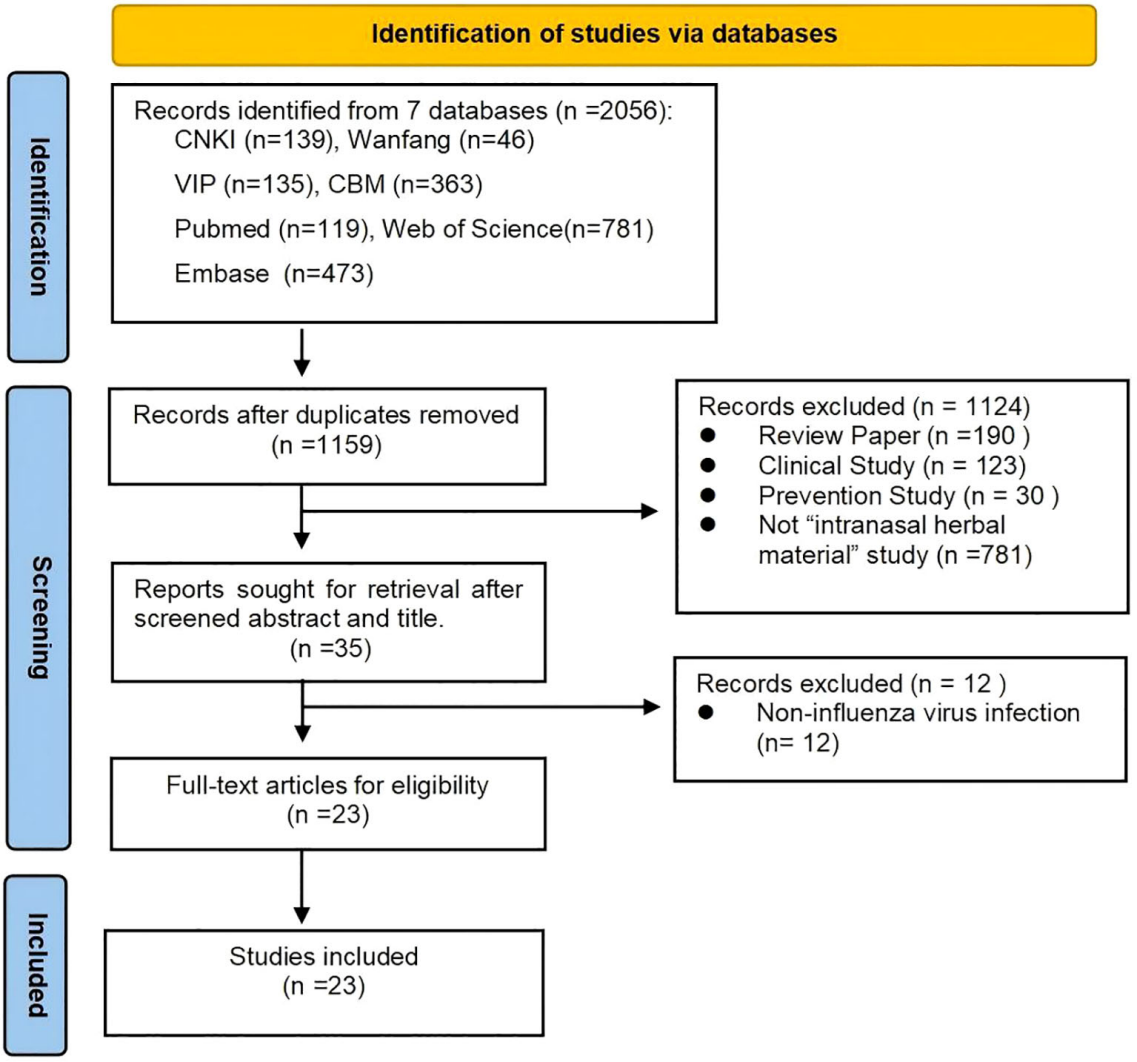
Flow chart of selection for included studies.

The systematic search identified a total of 2056 records from 7 databases: CNKI (139), Wanfang (46), VIP (135), CBM (363), PubMed (119), Web of Science (781), and Embase (473). After removing duplicates, 1159 records remained. After screening the titles and abstracts, 1124 records were excluded for reasons such as being review papers (n = 190), clinical studies (n = 123), prevention studies (n = 30), or not being “intranasal herbal material” studies (n = 781), leaving 35 reports for further retrieval. Following a full-text review, 12 records were excluded because they involved noninfluenza virus infection, and 23 eligible studies were ultimately included. The process of the literature search and selection is illustrated in **Figure 1**.

### 3.2 General characteristics of the included studies

A total of 23 eligible studies involving multiple influenza model animals and various natural products were included in this research. The sample sizes of the experimental groups ranged from 508–518, whereas those of the control groups ranged from 198–203; notably, two studies (18, 19) (Mao 2021, Yang 2022) did not report sample size data. All the studies established mouse models: 11 employed BALB/c mice (18–28), 5 used ICR mice (29–33), 4 used C57BL/6 mice (34–37), 2 used C57BL/6J mice (38, 39), and 1 used Kunming mice (40). The body weights of the mice ranged from 16–20 g (some studies did not report animal body weight data). All the influenza animal models were established via influenza virus inoculation. Most studies used H1N1 virus: 13 studies utilized PR8 virus for modeling, 8 used FM1 virus, and the remaining studies employed other virus strains, including H3N2 A/Aichi/2/68, H1N1 A/California/04/2009 (CA04), H9N2 A/Chicken/Guangdong/1996, H1N1 A/California/07/2009, and H3N2 A/Aichi/2/68. The routes of administration included intranasal instillation, aerosol inhalation, and nebulized inhalation, with the administration duration varying from several days to two weeks. The characteristics of these 23 eligible studies are detailed in **Table 1**.

**Table 1:** Basic characteristics of the included studies.

Author, year	Species (strain; age; weight)	Modeling Method (virus type, dose)	Sample size (T/M)		Intervention				Observation duration*	Outcomes
			T	M	Nature	Administration	Dose	Duration		
Hayashi 2007 (29)	Mouse (ICR; 4 weeks old; ≈17 g)	H1N1 PR8, 4LD_50_	25-30	25-30	Cinnamalde-hyde (CA)	Intranasal inoculation	250 μg	Once a day for 10 days	10 days	1. Survival rate 2. Body weight
			25-30			Natural volatilization	50mg/cage (5mice/cage)	The drug-containing solution was replaced once a day for 10 days		
									6 days	3. Viral titer
Serkedjieva 2008 (30)	Mice (ICR; 16–18 g)	H3N2 A/Aichi/2/68, 5–10 LD_50_	20 OR 3	20 OR 3	Polyphenolic complex (PC)	Aerosol	5.4 mg/ml (10 min)	Three doses, at 24h, 48h, and 72h post-infection	6 days or 14days	1. Survival rate (20 mice, 14days) 2. Viral titer (3 mice,6 days)
			20 OR 3					Four doses, at 0h, 24h, 48h, and 72h post-infection		
Smee 2011 (20)	Mice (BALB/c; 18–20 g)	H1N1 A/NWS/33, 4LD_50_	10	20	TheraMax	Intranasal administration	50 μl	Twice a day for 4 days	6 days	1. Survival rate 2. Viral titer
Theisen 2012 (21)	Mice (BALB/c; 7 weeks old)	H1N1 PR8, 4LD_50_	10	10	EPs^®^ 7630	Aerosol nebulization	5 mg/ml (10 min)	Three times a day for 10 days and single dose 10 min pre-viral infection	14 days	1. Survival rate 2. Viral titer 3. Body weight
		H1N1 PR8, 1LD_50_	10	10						
Haasbach 2014 (34)	Mice(C57BL/6; 6–8 weeks old)	H1N1 RB1, 5LD_50_	4	4	Ladania067	Aerosol nebulization	300 mg/6 ml	Twice a day for 5 days	10 days	1. Survival rate 2. Body weight
Kim 2015 (35)	Mice (C57BL/6 (H-2^b^); 6 weeks)	H1N1 PR8, 1LD_50_	10	10	Poly-gamma glutamate (γ-PGA)	Intranasal administration	100 μg	Single dose	14 days	1. Survival rate 2. Body weight
			3	3					3, 5, 7 days	3. Viral titer
			5	5					3 days	3. Inflammatory cytokines
		H1N1 CA04, 1LD_50_	10	10					14 days	1. Survival rate 2. Body weight
			3	3					3, 5, 7 days	3. Viral titer
			5	5					3 days	3. Inflammatory cytokines
Liu 2017 (22)	Mice (BALB/c; 20 ± 2g)	HIN1 FM1, 0.1mL per mouse	6	6	Volatile oil of Ganlu Xiaodu micropill	Intranasal instillation	0.04mL	Once a day for 6 days	7 days	1. Inflammatory cytokines
Liu 2018(1) (23)	Mice (BALB/c; 20 ± 2g)	HIN1 FM1, 0.1mL per mouse	6	6	Volatile oil of Ganlu Xiaodu micropill	Intranasal instillation	0.04mL	Once a day for 2 days	3 days	1. Lung index 2. Inflammatory cytokines
			6					Once a day for 6 days	7 days	
			6					Once a day for 9 days	10 days	
Liu 2018(2) (24)	Mice (BALB/c; 20 ± 2g)	HIN1 FM1, 0.1mL per mouse	6	6	Volatile oil of Ganlu Xiaodu micropill	Intranasal instillation	0.04mL	Once a day for 2 days	3 days	1. Inflammatory cytokines
			6					Once a day for 6 days	7 days	
			6					Once a day for 9 days	10 days	
Kuroki 2018 (38)	Mice (C57BL/6J; 8–10 weeks old)	H1N1 PR8, 10³ PFU	8	8	Lentinus edodes mycelia extract (LEM)	Intranasal administration	50μg	Single dose	14 days	1. Survival rate
Liu 2019 (25)	Mice (BALB/c; 20 ± 2g)	HIN1 FM1, 0.1mL per mouse	6	6	Volatile oil of Ganlu Xiaodu micropill	Intranasal instillation	0.04mL	Once a day for 2 days	3 days	1. Inflammatory cytokines
			6					Once a day for 6 days	7 days	
			6					Once a day for 9 days	10 days	
Yu 2019 (40)	Mice (Kunming; 4 weeks; 14.0 ± 1.0 g)	H1N1 PR8, 2LD_50_	10	10	Patchouli alcohol	Intranasal inoculation	20 μg/day	Once a day for 4 days	4 days	1. Viral titer 2. Inflammatory cytokines
			10				40 μg/day			
		H1N1 PR8, 4LD_50_	10				20 μg/day	Once a day for 7 days	14 days	3. Survival rate
			10				40 μg/day			
Zhang 2019 (31)	ICR mice	H1N1 PR8	20	20	Reduning	Nebulized inhalation	0.09 mL/kg (20 min)	Single dose	2 weeks	1. Survival rate
			20				0.04 mL/kg (10 min)			
			20				0.02 mL/kg (5 min)			
			20				0.01 mL/kg (2.5 min)			
		H1N1 FM1	20	20			0.09 mL/kg (20 min)		1 week	
			20				0.04 mL/kg (10 min)			
			20				0.02 mL/kg (5 min)			
			20				0.01 mL/kg (2.5 min)			
Mao 2021 (18)	Mice (BALB/c; 16–18 g)	H1N1 A/FM/1/47, 35μL	/	/	Luo Fu Shan Bai Cao Oil(LBO)	Ultrasonic atomization	100 μg/mL	5 days	/	1. Lung index 2. Inflammatory cytokines
			/	/			400 μg/mL			
Qiu 2021 (26)	Mice (BALB/c; 5–6 weeks old; 15.20 ± 0.93g)	H9N2 A/Chicken/Guangdong/1996, 2LD_50_	9	9	Shuanghuanglian	Aerosol	300 mg/mL, 0.5 mL/min (60 min)	Twice a day for 5 days	5 days	1. Viral titer 2. Inflammatory cytokines 3. Body weight
Sun 2021 (32)	Mice (ICR; 13-15g)	H1N1 PR8, 15LD_50_	10	10	Reduning	Aerosol	0.23 g/kg (20 min)	Once a day for 4 days	5 days	1. Viral titer 2. Lung index 3. Inflammatory cytokines
			10				0.12 g/kg (10 min)			
			10				0.06 g/kg (5 min)			
			10				0.03 g/kg (2.5 min)			
Joo 2022(1) (36)	Mice (C57BL/6; 6 weeks)	H1N1 A/Californ ia/07/2009 and H1N1 PR8, 10³ PFU	5	5	Elaeocarpus sylvestris Extract (ESE)	Intranasal administration	0.5 mg/kg	Three doses	14 days	1. Survival rate; 2. Body weight.
			5		1,2,3,4,6-penta-O-galloyl-β-D-glucose (PGG)					
			5		Geraniin (GE)					
Joo 2022(2) (37)	Mice (C57BL/6; 6–7 weeks old)	H1N1 A/California/07/2009, H1NI PR8 or H3N2 A/Aichi/2/68, 10³ PFU	5	5	Agrimonia pilosa and Galla rhois extracts (APRG64)	Intranasal administration	0.5 mg/kg	Three doses, at 10 min, 3 h, and 6 h post-infection	5 days	1. Viral titer
			5		Apigenin		0.25 mg/kg			
Yang 2022 (19)	Mice (BALB/c; 6–8 weeks)	H1N1 PR8, 3LD_50_	/	/	D-limonene	Intranasal inoculation	10 mg/kg	Once a day for 5 days	21 days	1. Survival rate 2. Viral titer 3. Body weight
							30 mg/kg			
			/	/	L-limonene		125 mg/kg			
							250 mg/kg			
Yang 2023 (27)	Mice (BALB/c; 6–8 weeks)	H1N1 PR8, 5LD_50_	6	6	Ginsenosides rk1 (G-rk1)	Intranasal administration	25 mg/kg	Initiated at 24 h post-infection, Once a day for 6 days	14 days	1. Survival rate
			6					Initiated at 48 h post-infection, Once a day for 6 days		
			6					Initiated at 72 h post-infection, Once a day for 6 days		
Bao 2024 (33)	Mice (ICR; 14 ± 1g)	H1N1 FM1, 15LD_50_	10	10	BD-77	Nebulized inhalation	75g/L (20 min)	Once a day for 4 days	5 days	1. Lung index
			10				75g/L (25 min)			
			10				37.5g/L (20 min)			
			10				37.5g/L (25 min)			
Mega 2024 (39)	Mice (C57BL/6J; 6–8 weeks)	H3N2 A virus X31, 10³ PFU	6	6	Enriched seaweed extract (ESE)	Intranasal administration	5 mg/kg	Once a day for 5 days	5 days	1. Viral titer
Wang 2024 (28)	Mice (BALB/c; 4 weeks old; 18–20 g).	H1N1 FM1, 15LD_50_	6	6	Patchouli	Nebulized inhalation	Converted from clinical dosage: 0.6 g/kg (crude herb) at 0.05 mL daily	Once a day for 5 days	5 days	1. Inflammatory cytokines
			6		Atractylodes					

*From virus infection.

### 3.3 Quality and risk assessment

**Figure 2:**
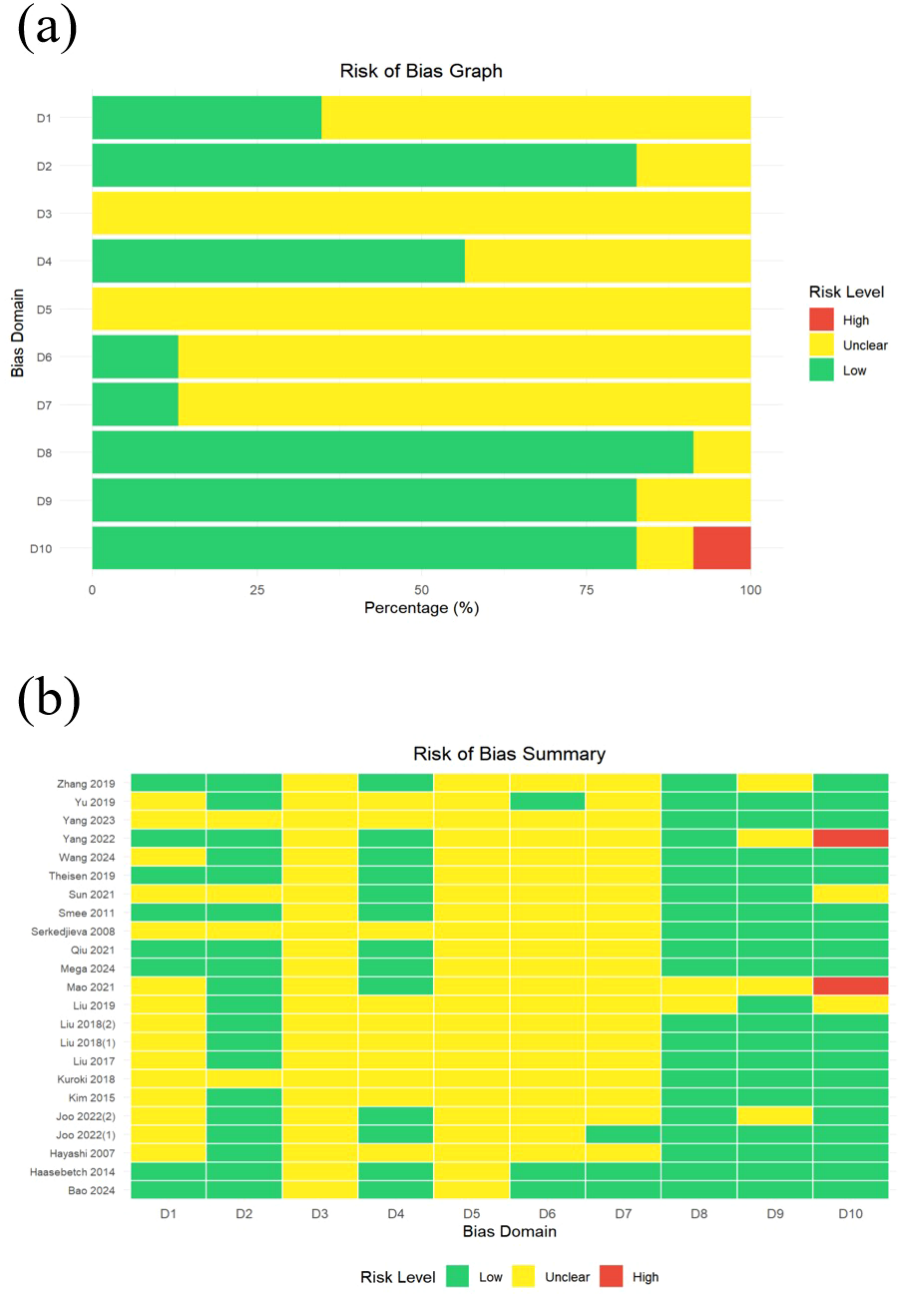
Risk of bias assessment for included studies. **(a)** Risk of bias graph. and **(b)** Risk of bias summary. The bias domains included are as follows: D1 refers to random sequence generation, D2 to baseline characteristics, D3 to allocation concealment, D4 to random animal housing, D5 to blinding of researchers, D6 to blinding of outcome assessors, D7 to random outcome assessment, D8 to incomplete outcome data, D9 to selective reporting, and D10 to other bias.

To assess the risk of bias in this meta-analysis involving 23 studies, key findings emerged. Overall, the reporting quality of the methodological details was inconsistent, leading to unclear methodological quality in multiple bias domains. Notably, 21 out of the 23 included studies were at low risk in the (D8) “incomplete outcome data” domain, which largely ensures the integrity of the outcome data and reduces the impact of attrition bias, whereas 2 studies were at unclear risk. In the (D2) “baseline characteristics” and (D9) “selective reporting” domains, 19 studies were rated as low risk for each. Specifically, the low risk in D2 excludes confounding biases arising from baseline imbalance, whereas that in D9 effectively minimizes concerns regarding reporting bias. In domains with prominent uncertainty, all the studies were rated as having unclear risk in both the (D3) “allocation concealment” and (D5) “blinding of researchers” domains; moreover, 20 out of the 23 studies had unclear risk in the (D6) “blinding of outcome assessors” and (D7) “random outcome assessment” domains. These results indicate that performance bias and detection bias cannot be fully excluded in these studies, primarily due to insufficient reporting of methodological details. In the “other bias” domain, 2 studies were at high risk (due to insufficient clarity regarding sample size), 19 studies were at low risk, and 2 studies were at unclear risk. In summary, although critical aspects such as outcome data completeness were relatively well controlled in most included studies, uncertainty persisted in the reporting of randomization and blinding-related methods, which should be noted when interpreting the meta-analysis results. The outcomes of the quality and risk assessment of the included studies are shown in **Figure 2**.

### 3.4 Effect of intranasal natural products on survival rate

**Figure 3:**
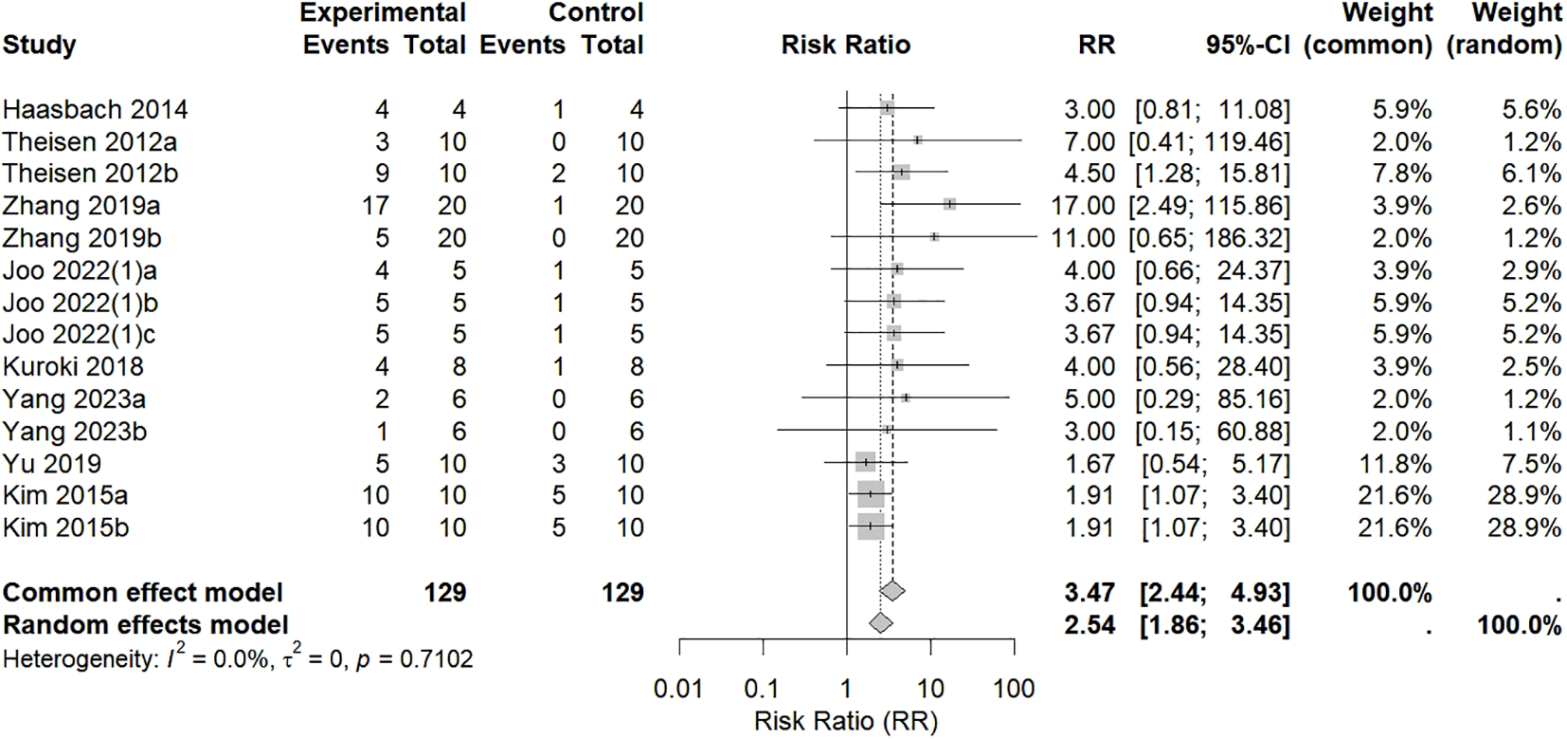
Effects of intranasal natural products on survival rate. Suffixes denote distinct experimental conditions within the same study: Theisen 2012a (4 LD_50_) and 2012b (1 LD_50_); Zhang 2019a (PR8) and 2019b (FM1); Joo 2022 (1)a (ESE), (1)b (PGG), and (1)c (GE); Yang 2023a (initiated at 24 h post-infection) and 2023b (initiated at 48 h post-infection); Kim 2015a (PR8) and 2015b (CA04).

A total of 8 studies (20, 21, 27, 31, 34–36, 38, 40) included 258 animal models, with experimental (n = 129) and control (n = 129) groups, and the combined analysis results are shown in **Figure 3**. There was no heterogeneity among the studies (*p* = 0.7102; I^2^ = 0%), so a common effects model was used. The results revealed that compared with the control treatment, the experimental intervention significantly improved survival (RR = 3.47, 95% CI: 2.44 to 4.93; *p* < 0.001).

It should be noted that this survival outcome is derived from studies with substantial differences in several key parameters, including: the use of diverse animal models (e.g., C57BL/6, BALB/c, Kunming, and ICR mice); variations in viral strains and virulence, involving multiple H1N1 subtypes such as PR8, FM1, and CA04, with challenge doses ranging from 1–5 LD_50_ or 10^3^ PFU; and heterogeneous administration protocols encompassing delivery methods, doses, and treatment courses. Against this backdrop of significant preclinical variability, the low statistical heterogeneity observed in the pooled analysis (I² = 0%) provides relatively consistent and preliminary preclinical evidence that intranasal administration of natural products may improve influenza survival rates. However, it must be emphasized that as key administration details remain unstandardized, the current evidence primarily supports the feasibility of this intervention direction. Its exact therapeutic efficacy and clinical translation potential require confirmation and elucidation through subsequent, more unified and targeted studies.

### 3.5 Effect of intranasal natural products on viral titer

**Figure 4:**
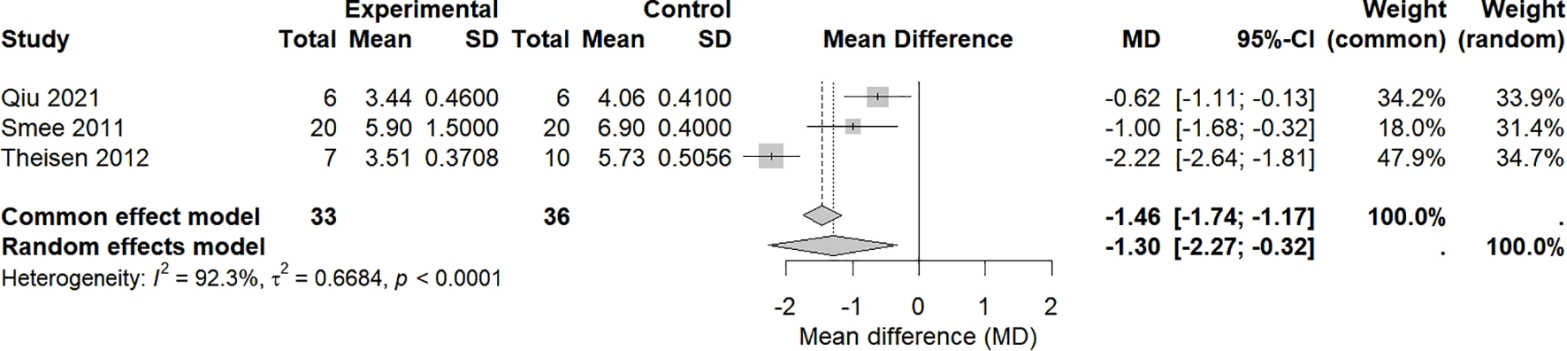
Effects of intranasal natural products on viral titer.

A total of 3 studies (20, 21, 26) included 69 animal models, with experimental (n = 33) and control (n = 36) groups, and the combined analysis results are shown in **Figure 4**. There was significant heterogeneity among the studies (*p* < 0.0001, I^2^ = 92.3%), so a random effects model was used. Meta-analysis revealed that the viral titer in the experimental group was significantly lower than that in the control group (MD = −1.30, 95% CI: −2.27–−0.32, *p* = 0.0092 < 0.01).

The studies included in this analysis all used BALB/c mice but differed in other key parameters: viral strains included various types such as H1N1 PR8, A/NWS/33, and H9N2; challenge doses ranged from 2–4 LD_50_. Particularly important is the significant variation in the assessment time points for viral titer (days 5, 6, and 14), an indicator highly sensitive to the infection progression. These factors are likely contributors to the observed heterogeneity. Due to the limited number of included studies (n=3), we were unable to perform subgroup analysis or meta-regression to quantify the specific contribution of each aforementioned factor to the heterogeneity. Therefore, this pooled effect size should be regarded merely as a qualitative description indicating a possible downward trend in viral titer, and this trend is subject to high uncertainty.

### 3.6 Effect of intranasal natural products on the lung index

**Figure 5:**
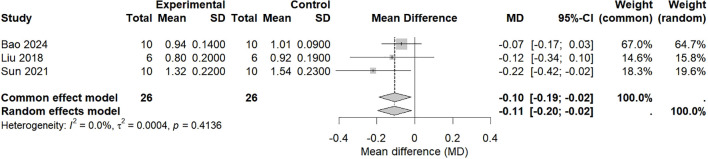
Effects of intranasal natural products on lung index.

A total of 3 studies (23, 32, 33) included 52 animal models, with experimental (n = 26) and control (n = 26) groups, and the combined analysis results are shown in **Figure 5**. There was no heterogeneity among the studies (*p* = 0.4136, I^2^ = 0%); therefore, a common effect model was used. Meta-analysis revealed that the lung index in the experimental group was significantly lower than that in the control group (MD =−0.10, 95% CI: −0.19–−0.02, *p* = 0.015 < 0.05).

The studies included in this lung index analysis exhibited variations across multiple parameters: the animal models involved ICR and BALB/c mice; virological challenges included different H1N1 subtypes such as PR8 and FM1, with challenge doses expressed as 15 LD_50_ or a fixed volume (0.1 mL per mouse); and routes of administration encompassed intranasal instillation, nebulization, and aerosol inhalation. Furthermore, considerable differences existed in operational details such as administration dose, treatment duration, and assessment time points across the studies. Against this backdrop, the low statistical heterogeneity (I² = 0%) observed primarily provides a preliminary preclinical evidence base. Its potential clinical value requires confirmation through more standardized and targeted future research.

### 3.7 Effect of intranasal natural products on weight

Eight studies involving 138 animals (experimental group/control group = 74/64) reported changes in body weight (**Table 2**). At both the lowest body weight point and the last detection time point, the body weight in the experimental group treated with natural products was greater than that in the control group. Four experiments from 2 studies (29, 36) clearly showed statistically significant differences (p < 0.05). Two studies (26, 39) reported no statistically significant differences (p > 0.05), and the results of Mega’s study indicated significant differences on the first and second days (not the lowest body weight point). The remaining 4 studies (19, 21, 34, 35) did not report p values, but the body weights of the experimental groups were consistently greater than those of the control groups. These results suggest that natural products affect the body weight of influenza-infected mice.

**Table 2:** Effects of intranasal natural products on weight.

Author, year	Modeling Method(Virus type, Dose)	Intervention			Lowest bodyweight point				Last observation time point				P
		Nature	Administration	Dose	T		M		T		M		
					Days	Body weight	Days	Body weight	Days	Body weight	Days	Body weight	
Hayashi 2007 (29)	H1N1 PR8, 4LD_50_	Cinnamalde-hyde (CA)	Intranasal inoculation	250 μg	7	114.6%^#+^	10	83.2%^#^	10	116.8%^#+^	△		<0.05
			Inhalation via natural volatilization	50 mg/cage (5 mice/cage)	10	110.1%^#+^			△				
Theisen 2012 (21)	H1N1 PR8, 4LD_50_	EPs^®^ 7630	Inhalation chamber connected to aerosol nebulizers	5 mg/ml (10 min)	8	78.7%^#^	7	75.8%^#^	14	93.1%^#^	△		/
Haasbach 2014 (34)	H1N1 RB1, 5LD_50_	Ladania067	Aerosol inhalation	300 mg/6 ml	6	75.1%^#^	7	69.7%^#^	10	77.8%^#^	△		/
Kim 2015 (35)	H1N1 PR8, 1LD_50_	Poly-gamma glutamate (γ-PGA)	Intranasal administration	100 μg	8	82.8%^#^	8	77.8%^#^	14	100.8%^#^	14	98.0%^#^	/
	H1N1 CA04, 1LD_50_				8	82.2%^#^	8	81.7%^#^	14	102.4%^#^	14	93.2%^#^	
Qiu 2021 (26)	H9N2 A/Chicken/Guangdong/1996, 2LD_50_	Shuanghuanglian	Aerosol inhalation	300 mg/mL, 0.5 mL/min (60 min)	5	90.9%	5	87.4%	△		△		>0.05
Joo 2022(1) (36)	H1N1 A/Californ ia/07/2009 and H1N1 PR8, 10³ pfu	Elaeocarpus sylvestris Extract (ESE)	Intranasal administration	0.5 mg/kg	8	79.2%^#^	8	72.8%^#^	14	97.2%^#^	△		<0.05
		1,2,3,4,6-penta-O-galloyl- β-D-glucose (PGG)			12	84.4%^#^			14	85.3%^#^			<0.05
		Geraniin (GE)			9	81.3%^#^			14	101.2%^#^			<0.001
Yang 2022 (19)	H1N1 PR8, 3LD_50_	D-limonene	Intranasal inoculation	10 mg/kg	6	72.0%^#^	8	65.4%^#^	21	102.3%^#^	△		/
				30 mg/kg	6	74.8%^#^			21	101.6%^#^			
		L-limonene		125 mg/kg	8	73.7%^#^	8	69.8%^#^	21	110.1%^#^	21	107.6%^#^	
				250 mg/kg	8	78.5%^#^			21	108.9%^#^			
Mega 2024 (39)	H3N2 A virus X31, 10³ PFU	Enriched seaweed extract	Intranasal administration	5 mg/kg	4	91.6%	4	91.3%	5	94.5%	5	92.7%	>0.05

#Data sourced from Origin 2024.

+Data compared with the control group of this study on the same day.

△Same as the data measured at the lowest point.

/P was no mentioned.

### 3.8 Effect of intranasal natural products on inflammatory cytokines

Nine studies reported the levels of inflammatory cytokines (**Table 3**). In studies investigating the levels of TNF-α, IL-6, IL-4, IL-5, and IL-1β, an overwhelming majority demonstrated a significant downregulatory effect (p < 0.05). In contrast, only in Kim’s study (35) did TNF-α exhibit an upregulatory effect (p < 0.05). Additionally, the TNF-α and IL-6 levels in a study on influenza-infected mice treated with Shuanghuanglian via aerosol inhalation (26), the IL-4 level in the serum and lung tissue of a group treated with intranasal instillation of the volatile oil of Ganlu Xiaodu Micropill (24) (Liu 2018 (2)), and the IL-1β level in the Atractylodes group in Wang et al.’s study (Wang 2024) all failed to clearly decrease (p > 0.05). In studies examining the levels of IFN-α, IFN-β, IFN-γ, and IL-12, an overwhelming majority showed a significant upregulatory effect (p < 0.05). In contrast, only IFN-γ in Wang et al.’s study displayed a downregulatory effect (p < 0.05). Moreover, IFN-γ in the serum samples from Liu et al.’s study [Liu 2018 (2)] failed to exhibit a clear upregulatory trend (p > 0.05). Despite variations in sampling sites (serum, lung tissue, or alveolar lavage fluid), these results consistently confirm the regulatory effect of natural products on inflammatory cytokines in influenza-infected mice.

**Table 3:** Effects of intranasal natural products on inflammatory cytokines.

Inflammatory cytokines	Author, year	Treatment group	*P*	Trend	Source	Unit
TNF-α	Wang 2024a (28)	18.93	<0.01	↓	Alveolar lavage fluid	pg/ml
	Wang 2024b (28)	6	<0.05	↓		pg/ml
	Liu 2018(1) (36)	28.94	<0.05	↓	Serum	pg/ml
	Sun 2021 (32)	113.98	<0.01	↓	Lung tissue	pg/ml
	Qiu 2021 (26)	941.36^#^	>0.05	↓	Lung tissue	pg/g
	Kim 2015a (35)	4.44^#^	<0.05	↑	Lung tissue	Fold of control
	Kim 2015b (35)	4.67^#^	<0.05	↑		Fold of control
IL-6	Liu 2018(1) (23)	56.62	<0.05	↓	Serum	pg/ml
	Sun 2021 (32)	78.38	<0.01	↓	Lung tissue	pg/ml
	Qiu 2021 (26)	4582.48^#^	>0.05	↓	Lung tissue	pg/g
	Mao 2021 (18)	8.77^#^	<0.05	↓	Serum	pg/ml
IL-4	Liu 2018(2)a (24)	97.14	>0.05	↓	Serum	pg/ml
	Liu 2018(2)b (24)	1568.14	>0.05	↓	Lung tissue	ng/g
IL-5	Liu 2019 (25)	244.66	<0.05	↓	Serum	pg/ml
IL-1β	Mao 2021 (18)	7.77^#^	<0.001	↓	Serum	pg/ml
	Wang 2024a (28)	0.14	<0.01	↓	Alveolar lavage fluid	pg/ml
	Wang 2024b (28)	0.17	>0.05	↓		pg/ml
IFN-α	Liu 2017 (22)	21.81	<0.05	↑	Serum	pg/ml
IFN-β	Kim 2015a (35)	6.2^#^	<0.01	↑	Lung tissue	Fold of control
	Kim 2015b (35)	6.6^#^	<0.01	↑		Fold of control
	Liu 2017 (22)	155.17	<0.05	↑	Serum	pg/ml
IFN-γ	Liu 2018(2)a (24)	21.94	>0.05	↑	Serum	pg/ml
	Liu 2018(2)b (24)	344.73	<0.05	↑	Lung tissue	ng/g
	Wang 2024a (28)	15.36	<0.01	↓	Alveolar lavage fluid	pg/ml
	Wang 2024b (28)	20.61	<0.01	↓		pg/ml
IL-12	Kim 2015a (35)	7.15^#^	<0.01	↑	Lung tissue	Fold of control
	Kim 2015b (35)	7.16^#^	<0.01	↑		Fold of control

#Data sourced from Origin 2024.

Wang 2024a: Patchoul intervention; Wang 2024b: Atractylodes intervention; Kim 2015a: PR8 virus modeling; Kim 2015b: CA04 virus modeling; Liu 2018(2)a: Source from serum; Liu 2018(2)b: Source from Lung tissue. "↓" indicates a downregulatory effect. "↑" indicates an upregulatory effect.

### 3.9 Sensitivity analysis

**Figure 6:**
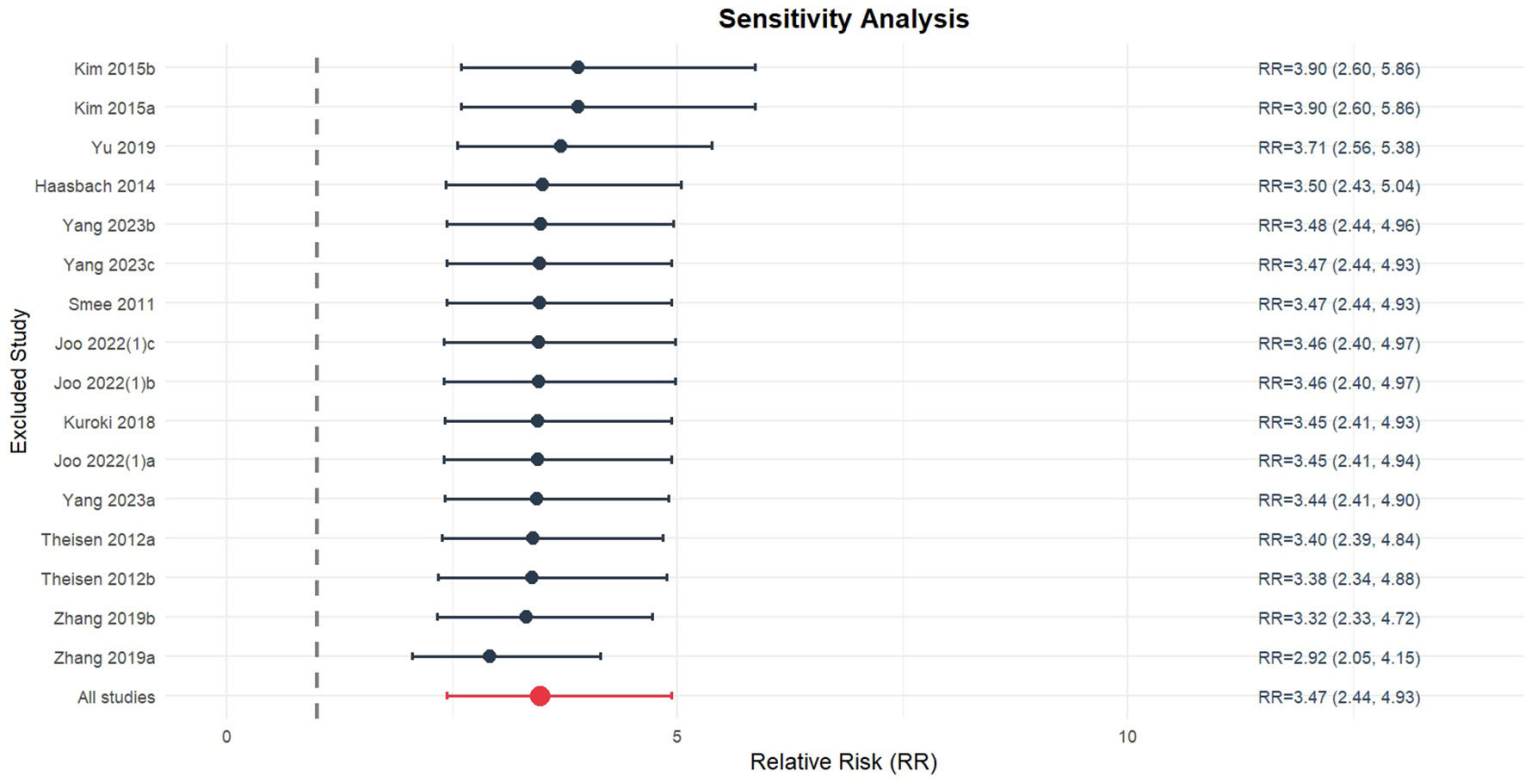
Sensitivity analysis of efficacy of intranasal natural products on survival rate.

Sensitivity analysis was conducted to evaluate the impact of individual studies on survival rate. Each study was sequentially excluded using a stepwise approach, and the pooled effect size of the remaining studies was recalculated. The results demonstrated that the 95% confidence interval (CI) of the pooled effect size did not change significantly regardless of which single study was excluded (**Figure 6**). These findings indicated that the analysis results were not strongly dependent on any individual study and that the conclusions were highly robust. After each study was excluded one by one, the pooled effect size of the remaining studies remained consistent and did not significantly change. Even when studies with relatively high RR—such as Kim 2015a and Kim 2015b (both with an RR of 3.03)—or those with wide CIs (e.g., Zhang 2019a and Zhang 2019b) were excluded, the pooled effect size remained stable within a similar range. Notably, the 95% CI never included 1.00, confirming that the effect of the intervention was statistically significant. These findings suggest that the intervention’s effect on reducing the incidence of the target event has high stability and reliability. Although some studies may exhibit relatively large effect sizes or wide CIs, their impact on the overall analysis results is limited. Therefore, it can be concluded that the effect of the intervention is consistent across different studies, which supports its effectiveness and reliability in practical applications.

### 3.10 Publication bias analysis

**Figure 7:**
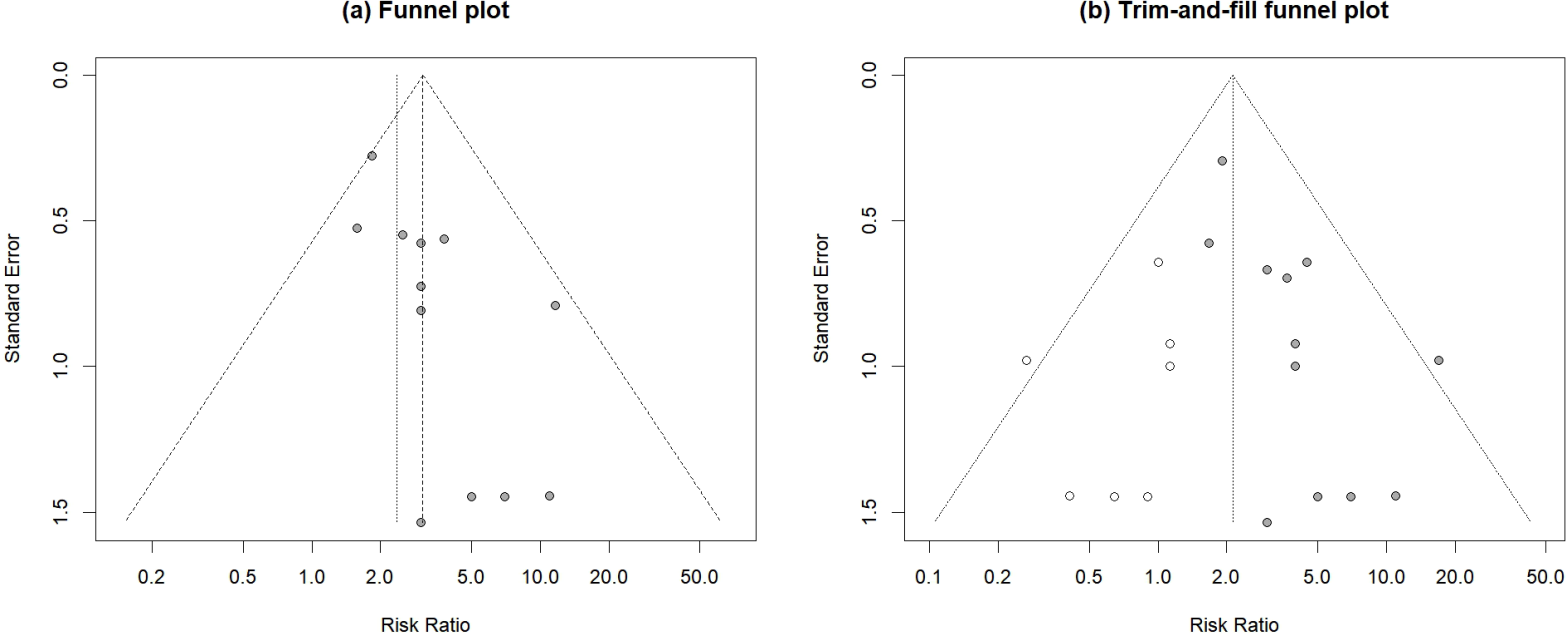
Funnel plot for publication bias assessment of survival rate. **(a)** Funnel plot. and **(b)** Trim -and-fill funnel plot. The gray solid circles represent the 14 original studies included. The white hollow circles represent 7 potentially missing studies imputed by the trim-and-fill algorithm on the left side of the plot.

Publication bias was assessed using funnel plots, Egger’ s regression test, and the trim-and-fill method. Visual inspection of the funnel plot (**Figure 7a**) revealed asymmetry in the distribution of the original studies (gray dots), suggesting potential publication bias. This asymmetry was statistically confirmed by Egger’ s test (*p* = 0.0009 < 0.05). To adjust for this bias, was performed using the trim−and−fill method. As shown in **Figure 7b**, the algorithm imputed seven potentially missing studies (white hollow circles) on the left side of the funnel plot. After incorporating these imputed studies, the pooled RR calculated with a random−effects model was 2.12 (95% CI: 1.60–2.82, *p* < 0.0001), which remained consistent with the original analysis in both direction and statistical significance. Between−study heterogeneity was very low (*I²* = 0.8%). These findings indicate that, although statistical evidence of publication bias exists, it did not alter the main conclusion of this meta−analysis.

## 4 Discussion

**Figure 8:**
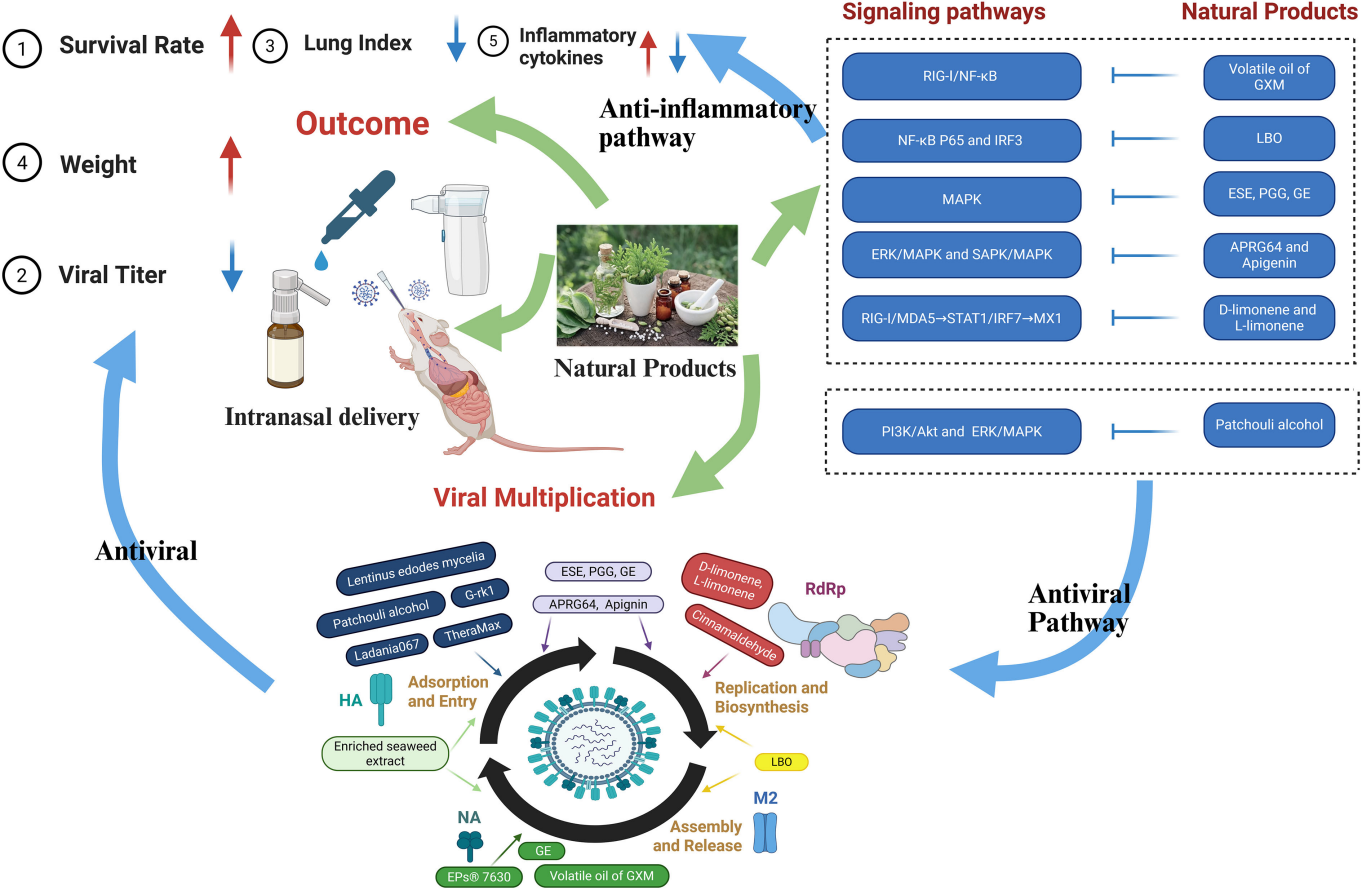
Mechanisms of intranasal natural products against influenza.

In this study, a systematic review and meta-analysis were conducted to evaluate the efficacy of natural products in treating influenza via intranasal administration. The 23 studies included exhibited significant differences in the selection of intervention drugs, as well as obvious heterogeneity in aspects such as intranasal delivery routes, treatment doses, durations of treatment, and observation durations. Despite the aforementioned heterogeneity, most of the included studies demonstrated that intranasally administered natural products exerted significant positive effects on influenza models, specifically by increasing the survival rate, reducing viral titers, decreasing the lung index, improving body weight, and regulating the levels of inflammatory cytokines. Furthermore, we also found that natural products exerted anti-influenza effects by inhibiting viral multiplication, regulating inflammatory cytokines, and modulating multiple signaling pathways (**Figure 8**).

### 4.1 Multistage mechanisms for inhibiting viral multiplication by natural products

Natural products can effectively block viral absorption and entry. Ladania067 interacts with the invading pathogen; TheraMax (20) blocks viral absorption, APRG64 and apigenin (37) inhibit attachment and entry; patchouli alcohol (40) inactivates viruses postadsorption; and Lentinus edodes extract (38) acts on early infection, possibly by targeting entry. Additionally, ESE (36) and its main components—1,2,3,4,6-penta-O-galloyl-β-D-glucose (PGG) and geraniin (GE)—as well as EPs^®^7630 (21), enriched seaweed extract (39) and G-rk1 (27), block viral absorption on cells by acting on hemagglutinin (HA). Hemagglutinin (HA) is a key glycoprotein on the surface of IAV/IBV and plays an irreplaceable role in the viral adsorption process (41, 42). It is not only the core target of antiviral strategies but also the main focus of currently licensed influenza vaccine immunization (43); furthermore, high-titer HA-specific antibodies are directly associated with the prevention of IAV/IBV infections and protection against influenza, which requires medical care (44), highlighting its core importance in anti-influenza virus responses.

Natural products can directly interfere with influenza virus replication through multiple mechanisms. ESE, PGG, and GE (36) significantly inhibit the polymerase activity of IAV; LBO (18), cinnamaldehyde (29), and patchouli alcohol (40) block the replication process by suppressing the expression of virus-related proteins. Notably, D-limonene and L-limonene (the main active components of Maqian) (19), along with APRG64 and apigenin (37), specifically inhibit the activity of RNA-dependent RNA polymerase (RdRp). RdRp inhibitors have emerged as a novel strategy against influenza viral infections, and the FDA’s approval of baloxavir marboxil in 2018 further confirmed the importance of this drug class (45). As a key enzyme for viral replication, RdRp has a highly conserved structure across all influenza virus strains and subtypes (46, 47). Additionally, natural products, including epigallocatechin-3-O-gallate and licoflavone B, have been shown to exert anti-influenza effects by targeting RdRp (48, 49). Thus, inhibiting viral replication by targeting RdRp is expected to be an effective approach for influenza treatment.

Natural products primarily inhibit influenza virus release by targeting key viral proteins, including matrix 2 ion channel (M2) and neuraminidase (NA). LBO (18) inhibits viral release by downregulating the matrix 2 ion channel (M2), whereas enriched seaweed extract (39), EPs^®^7630 (21), and GE (36) target the NA to block progeny viral release, thereby reducing viral spread and infection to surrounding new cells. Neuraminidase (NA), a glycoprotein on influenza virus particles, promotes the release of new viral particles from infected cells by cleaving sialic acid residues in glycoproteins and glycolipids (50, 51). Widely used antiviral drugs such as oseltamivir and zanamivir are neuraminidase inhibitors (52), and studies have proposed identifying natural products as influenza NA inhibitors via in silico screening, *in vitro* validation, and molecular dynamic simulation (53).

### 4.2 Dual modulation of inflammatory cytokines by natural products

Natural products exhibit remarkable anti-inflammatory properties, primarily through the modulation of dysregulated inflammatory signaling networks—a mechanism crucial for mitigating excessive inflammation associated with viral infections. By targeting key proinflammatory cytokines, including tumor necrosis factor-alpha (TNF-α), interleukin-6 (IL-6), IL-4, IL-5, and IL-1β, natural products effectively attenuate the initiation and propagation of inflammatory cascades. The downregulation of these cytokines not only reduces tissue damage, as evidenced by the alleviation of influenza-induced pulmonary inflammation, but also minimizes the risk of cytokine storms, a potentially life-threatening hyperinflammatory response (54). Notably, the natural medicinal herb Scutellaria baicalensis Georgi has been identified as a particularly promising candidate therapeutic for preventing infection-related cytokine storms (55). Similarly, the natural product Lentinan has been shown to mitigate cytokine storms induced by influenza virus infection (56).

### 4.3 Balanced regulation of signaling pathways by natural products

Natural products exert anti-influenza effects through targeted regulation of dysregulated signaling pathways, resulting in distinct antiviral and anti-inflammatory mechanisms. In terms of antiviral activity, patchouli alcohol (40) inhibits influenza virus invasion and subsequent replication by suppressing the PI3K/Akt and ERK/MAPK pathways. Viral survival relies on the evolution of strategies to modulate host cellular signaling pathways, particularly those governing cell death and survival (57). Moreover, influenza virus replication is dependent on host cellular mechanisms involved in cell survival, such as the ERK and PI3K–Akt pathways (58). Among these, PI3K acts as a key regulator of multiple signal transduction pathways, targeting several downstream effectors, including Akt and ERK, to modulate a variety of cellular events (59).

With respect to anti-inflammatory effects, multiple natural products converge on core proinflammatory pathways. Volatile oil from GXM (22) inhibits excessive activation of the RIG-I/NF-κB pathway, whereas LBO (18) targets NF-κB P65 and IRF3 activation—both of which reduce proinflammatory cytokine release. ESE (36), APRG64, and apigenin (37) block the activation of the MAPK pathway (including ERK/SAPK branches), a key hub for propagating inflammatory cascades. Notably, D-limonene and L-limonene (19) correct overactivation of the RIG-I/MDA5→STAT1/IRF7→MX1 pathway, thereby balancing antiviral immunity and inflammatory responses. This multipathway modulation circumvents the single-target limitations of synthetic drugs; by regulating both the viral replication machinery and host inflammatory responses, natural products offer promising therapeutic strategies against influenza. Consistent with these findings, studies by Tie et al. have shown that natural products exert multitargeted, low-toxicity protective effects against viral pneumonia—mainly caused by influenza viruses—by modulating inflammation and oxidative stress-related pathways, further underscoring their potential as promising therapeutic strategies for treating viral respiratory diseases (60).

### 4.4 Limitations

This meta-analysis of natural products for influenza via intranasal administration has significant limitations that affect the robustness of the results. First, excessive confounding factors prevail. The analysis encompasses diverse natural product types, including single compounds, multicomponent prescriptions, and herbal extracts. Intranasal administration methods vary widely, ranging from nebulization and intranasal instillation to aerosol inhalation. Moreover, the studies involve nonunified influenza virus strains such as H1N1 and H3N2, all of which increase study heterogeneity. Notably, for survival rate and lung index, the pooled analysis showed no statistical heterogeneity (I²= 0%) despite the substantial experimental heterogeneity described above. This situation, in which included studies present substantial differences yet still show I² = 0%, has also been reported in a preclinical meta-analysis by Kalantari et al. (61). Thus, we should not rely solely on the I² statistic when interpreting heterogeneity. Over-reliance on this single measure may lead to overestimation or underestimation of the pooled results for these two outcomes (62). Second, study design flaws exist. Most studies lack positive controls such as oseltamivir and rely solely on blank controls. The lack of uniformity in outcome indicators across studies poses a significant challenge to data synthesis. Additionally, observation durations and sample sizes vary, reducing statistical power. Third, reporting inadequacies are evident. Many studies fail to provide details on randomization and blinding, introducing risks of selection and performance bias. Incomplete descriptions of administration methods and drug doses, along with inconsistent units for outcome indicators, impede data synthesis and reproducibility. Finally, potential publication bias should be considered. The funnel plot exhibited asymmetry, which may stem from variations in experimental conditions, procedures, and personnel across studies, or could indicate a systematic underrepresentation of smaller studies with neutral or negative findings. Although statistical adjustments were applied, the possibility remains that unpublished negative results, which are crucial for a balanced evidence base, were missed, potentially leading to an overestimation of the pooled effect size.

### 4.5 Future research directions

To address the limitations of current studies and promote the clinical translation of natural products for influenza via intranasal administration, future research should focus on several key directions. First, it is urgent to establish standardized guidelines. For different stages of influenza, including acute infection and recovery, unified influenza virus strains should be used. Corresponding consistent outcome indicators, such as the viral clearance rate and inflammatory cytokine levels, along with appropriate observation durations, should be formulated. Moreover, sample sizes need to be expanded to increase statistical reliability, and positive controls, e.g., clinical antiviral drugs such as oseltamivir, should be added to better evaluate the relative efficacy of natural products.

Second, studies on dosage form transformation and administration method optimization should be strengthened. It has been reported that converting injectable natural products into atomized inhalation formulations can increase the local drug concentration in the respiratory tract and reduce systemic side effects, which deserves further clinical verification (32). Current research indicates that systematic comparative studies on the efficacy and safety of different intranasal administration methods, including nebulization and intranasal instillation, should be conducted to screen the most suitable administration modes for specific natural products (31).

Moreover, in-depth exploration of the antiviral mechanism of existing natural products is essential. By clarifying their regulatory effects on viral replication, inflammatory signaling pathways, and other processes, new action targets can be identified to provide theoretical support for the development of high-efficiency natural products. Additionally, research on the preventive effect of natural products against influenza should not be neglected. Existing studies suggest that exploring the role of intranasally administered natural products in enhancing respiratory mucosal immunity and inhibiting initial viral attachment can expand their application from treatment to prevention, further promoting their public health value in influenza control (63, 64).

## 5 Conclusion

Natural products can improve the survival rate, reduce the pulmonary viral load, and alleviate pneumonic lesions, with positive trends in restoring postinfection body weight and regulating inflammatory cytokines. Their anti-influenza effects may involve direct inhibition of viral replication, anti-inflammation, and signaling pathway regulation, supporting their potential as therapeutic candidates for influenza. However, to accurately assess their anti-influenza properties and provide sufficient preclinical evidence for clinical translation, broader, longer-duration, high-quality preclinical studies are needed. Future research should standardize animal experiment protocols and reporting to enhance evidence quality.

## Data Availability

The original contributions presented in the study are included in the article/**Supplementary Material**. Further inquiries can be directed to the corresponding author.
