# Supplementary material for: Intranasal natural products for influenza treatment: a systematic review and meta-analysis of preclinical studies
Source: Front Immunol. 2026 Mar 10;17:1758831. doi: 10.3389/fimmu.2026.1758831 (PMC13008708; doi:10.3389/fimmu.2026.1758831)
Supplement: Supplementary file 1 [file Table1.docx]

**Supplementary Table 1.** Search strategy

|  | Search strategy |
| --- | --- |
| PubMed  (N = 119) | 1. "influenza, human"[MeSH Terms] OR "Influenza A virus"[MeSH Terms] OR "Influenza B virus"[MeSH Terms] OR "Orthomyxoviridae"[MeSH Terms] OR "influenza"[Title/Abstract] OR "flu"[Title/Abstract] 2. "Biological Products"[MeSH Terms] OR "Phytochemicals"[MeSH Terms] OR ("drugs, chinese herbal"[MeSH Terms] OR "Herbal Medicine"[MeSH Terms] OR "Phytotherapy"[MeSH Terms] OR "Plant Extracts"[MeSH Terms] OR "medicine, traditional"[MeSH Terms] OR "medicine, east asian traditional"[MeSH Terms] OR "medicine, korean traditional"[MeSH Terms] OR "medicine, tibetan traditional"[MeSH Terms] OR "medicine, mongolian traditional"[MeSH Terms] OR "medicine, african traditional"[MeSH Terms] OR "medicine, chinese traditional"[MeSH Terms] OR "medicine, iranian traditional"[MeSH Terms] OR "oils, volatile"[MeSH Terms] "Perfume"[MeSH Terms] OR "biologic* product*"[Title/Abstract] OR "natural product*"[Title/Abstract] OR "biopharmaceutical*"[Title/Abstract] OR "biologic*"[Title/Abstract] OR "Plant Derived Compound"[Title/Abstract] OR "Plant Bioactive Compound"[Title/Abstract] OR "Plant Derived Chemical"[Title/Abstract] OR "Phytochemical"[Title/Abstract] OR "Phytonutrient"[Title/Abstract] OR "Pharmacognosy"[Title/Abstract] OR "Ethnobotany"[Title/Abstract] OR "Ethnopharmacology"[Title/Abstract] OR "Traditional Medicine"[Title/Abstract] OR "medicine east asian traditional"[Title/Abstract] OR "medicine korean traditional"[Title/Abstract] OR "medicine african traditional"[Title/Abstract] OR "Traditional Chinese Medicine"[Title/Abstract] OR "medicine iranian traditional"[Title/Abstract] OR "Perfume"[Title/Abstract] OR "Volatile Oil"[Title/Abstract] OR "Essential Oil"[Title/Abstract] 3. "administration, intranasal"[MeSH Terms] OR "Nasal Absorption"[MeSH Terms] OR "Nebulizers and Vaporizers"[MeSH Terms] OR "administration, inhalation"[MeSH Terms] OR "Nasal Sprays"[MeSH Terms] OR "Aromatherapy"[MeSH Terms] OR "intranasal administration"[Title/Abstract] OR "intranasal drug administration"[Title/Abstract] OR "nasal administration"[Title/Abstract] OR "atomizer*"[Title/Abstract] OR "inhaler*"[Title/Abstract] OR "nebulizer*"[Title/Abstract] OR "vaporizer*"[Title/Abstract] OR "Inhalation"[Title/Abstract] OR "Aerosol"[Title/Abstract] OR "respiratory drug administration"[Title/Abstract] OR "Nasal Spray"[Title/Abstract] OR "Nasal Mist"[Title/Abstract] OR "Intranasal instillation"[Title/Abstract] OR "aroma*"[Title/Abstract] 4. "Animals"[MeSH Terms] OR "Animal Experimentation"[MeSH Terms] OR "Animals"[Title/Abstract] OR "Animal Experimentation"[Title/Abstract] OR "rat"[Title/Abstract] OR "rats"[Title/Abstract] OR "mouse"[Title/Abstract] OR "mice"[Title/Abstract] OR "swine"[Title/Abstract] OR "porcine"[Title/Abstract] OR "murine"[Title/Abstract] OR "sheep"[Title/Abstract] OR "lambs"[Title/Abstract] OR "pigs"[Title/Abstract] OR "piglets"[Title/Abstract] OR "rabbit"[Title/Abstract] OR "rabbits"[Title/Abstract] OR "cat"[Title/Abstract] OR "cats"[Title/Abstract] OR "dog"[Title/Abstract] OR "dogs"[Title/Abstract] OR "cattle"[Title/Abstract] OR "bovine"[Title/Abstract] OR "monkey"[Title/Abstract] OR "monkeys"[Title/Abstract] OR "trout"[Title/Abstract] OR "marmoset"[Title/Abstract] OR "bird*"[Title/Abstract] OR "avian"[Title/Abstract] 5. #1 AND #2 AND #3 AND #4 |
| Embase  (N = 473) | 1. 'influenza'/exp AND [embase]/lim 2. ('biological product'/exp OR 'medicinal plant'/exp OR 'traditional medicine'/exp OR 'perfume'/exp OR 'aromatherapy'/exp OR 'aroma'/exp OR 'herbaceous agent'/exp OR 'phytochemical'/exp OR 'plant extract'/exp OR 'essential oil'/exp) AND [embase]/lim OR ('biologic':ab,ti OR 'biologic agent':ab,ti OR 'biologic product':ab,ti OR 'biological':ab,ti OR 'biological agent':ab,ti OR 'biological product':ab,ti OR 'biologicals':ab,ti OR 'biologics':ab,ti OR 'natural compound':ab,ti OR 'natural product':ab,ti OR 'ethnomedicinal plant':ab,ti OR 'medicinal plant':ab,ti OR 'plants, medicinal':ab,ti OR 'phytochemical':ab,ti OR 'phytopharmaceutical':ab,ti OR 'plant-derived compound':ab,ti OR 'drugs, chinese herbal':ab,ti OR 'herbaceous drug':ab,ti OR 'herbal agent':ab,ti OR 'herbal drug':ab,ti OR 'herbal medicinal product':ab,ti OR 'ethnomedicine':ab,ti OR 'folk medicine':ab,ti OR 'traditional medicine':ab,ti OR 'volatile oil':ab,ti OR 'essential oil':ab,ti OR 'perfume':ab,ti OR 'aroma*':ab,ti) AND [embase]/lim 3. #1:('intranasal drug administration'/exp OR 'intranasal drug administration' OR 'inhalational drug administration'/exp OR 'inhalational drug administration' OR 'nebulizer'/exp OR 'nebulizer') AND or perfume:ab AND [embase]/lim OR ('intranasal administration':ab,ti OR 'intranasal drug administration':ab,ti OR 'intranasal instillation':ab,ti OR 'intranasal medication':ab,ti OR 'nasal administration':ab,ti OR 'nasal drug administration':ab,ti OR 'nasal instillation':ab,ti OR 'nasal medication':ab,ti OR 'nasal absorption':ab,ti OR 'inhalation administration':ab,ti OR 'inhalation drug administration':ab,ti OR 'inhalation medication':ab,ti OR 'inhalation therapy':ab,ti OR 'inhalation treatment':ab,ti OR 'inhaled drug':ab,ti OR 'aerosol therapy':ab,ti OR 'drug inhalation':ab,ti OR 'vapor therapy':ab,ti OR 'atomizer':ab,ti OR 'nebuliser':ab,ti OR 'nebulizer':ab,ti OR 'nebulizers and vaporizers':ab,ti OR 'vaporiser':ab,ti OR 'vaporizer':ab,ti OR 'inhalation device':ab,ti OR 'inhalator':ab,ti OR 'inhaler':ab,ti OR 'aerosol exposure':ab,ti OR 'aerosol inhalation':ab,ti OR 'aerosolization':ab,ti OR 'aerosols':ab,ti OR 'pressurized aerosol':ab,ti OR 'spray':ab,ti OR 'aerosol':ab,ti) AND [embase]/lim 4. 'animal'/exp OR 'animal experiment'/exp AND [embase]/lim 5. #1 AND #2 AND #3 AND #4 |
| Web of SCI  (N = 781) | 1. TS=(influenza, human OR Orthomyxoviridae OR Influenza A virus OR Influenza B virus OR influenza OR flu) 2. TS=(biologic* product* OR natural product* OR biopharmaceutical* OR biologic* OR Plant Derived Compound OR Plant Bioactive Compound OR Plant Derived Chemical OR Phytochemical OR Phytonutrient OR Pharmacognosy OR Ethnobotany OR Ethnopharmacology OR Traditional Medicine OR medicine east asian traditional OR medicine korean traditional OR medicine african traditional OR Traditional Chinese Medicine OR medicine iranian traditional OR Perfume OR Volatile Oil OR Essential Oil) 3. TS=(intranasal administration OR intranasal drug administration OR Nasal Sprays OR nasal administration OR atomizer* OR inhaler* OR nebulizer* OR vaporizer* OR Inhalation OR Aerosol OR respiratory drug administration OR Nasal Mist OR Intranasal instillation OR aroma*) 4. TS=(animal or animals or pisces or fish or fishes or catfish or catfishes or sheatfish or silurus or arius or heteropneustes or clarias or gariepinus or fathead minnow or fathead minnows or pimephales or promelas or cichlidae or trout or trouts or char or chars or salvelinus or salmo or oncorhynchus or guppy or guppies or millionfish or poecilia or goldfish or goldfishes or carassius or auratus or mullet or mullets or mugil or curema or shark or sharks or cod or cods or gadus or morhua or carp or carps or cyprinus or carpio or killifish or eel or eels or anguilla or zander or sander or lucioperca or stizostedion or turbot or turbots or psetta or flatfish or flatfishes or plaice or pleuronectes or platessa or tilapia or tilapias or oreochromis or sarotherodon or common sole or dover sole or solea or zebrafish or zebrafishes or danio or rerio or seabass or dicentrarchus or labrax or morone or lamprey or lampreys or petromyzon or pumpkinseed or pumpkinseeds or lepomis or gibbosus or herring or clupea or harengus or amphibia or amphibian or amphibians or anura or salientia or frog or frogs or rana or toad or toads or bufo or xenopus or laevis or bombina or epidalea or calamita or salamander or salamanders or newt or newts or triturus or reptilia or reptile or reptiles or bearded dragon or pogona or vitticeps or iguana or iguanas or lizard or lizards or anguis fragilis or turtle or turtles or snakes or snake or aves or bird or birds or quail or quails or coturnix or bobwhite or colinus or virginianus or poultry or poultries or fowl or fowls or chicken or chickens or gallus or zebra finch or taeniopygia or guttata or canary or canaries or serinus or canaria or parakeet or parakeets or grasskeet or parrot or parrots or psittacine or psittacines or shelduck or tadorna or goose or geese or branta or leucopsis or woodlark or lullula or flycatcher or ficedula or hypoleuca or dove or doves or geopelia or cuneata or duck or ducks or greylag or graylag or anser or harrier or circus pygargus or red knot or great knot or calidris or canutus or godwit or limosa or lapponica or meleagris or gallopavo or jackdaw or corvus or monedula or ruff or philomachus or pugnax or lapwing or peewit or plover or vanellus or swan or cygnus or columbianus or bewickii or gull or chroicocephalus or ridibundus or albifrons or great tit or parus or aythya or fuligula or streptopelia or risoria or spoonbill or platalea or leucorodia or blackbird or turdus or merula or blue tit or cyanistes or pigeon or pigeons or columba or pintail or anas or starling or sturnus or owl or athene noctua or pochard or ferina or cockatiel or nymphicus or hollandicus or skylark or alauda or tern or sterna or teal or crecca or oystercatcher or haematopus or ostralegus or shrew or shrews or sorex or araneus or crocidura or russula or european mole or talpa or chiroptera or bat or bats or eptesicus or serotinus or myotis or dasycneme or daubentonii or pipistrelle or pipistrellus or cat or cats or felis or catus or feline or dog or dogs or canis or canine or canines or otter or otters or lutra or badger or badgers or meles or fitchew or fitch or foumart or foulmart or ferrets or ferret or polecat or polecats or mustela or putorius or weasel or weasels or fox or foxes or vulpes or common seal or phoca or vitulina or grey seal or halichoerus or horse or horses or equus or equine or equidae or donkey or donkeys or mule or mules or pig or pigs or swine or swines or hog or hogs or boar or boars or porcine or piglet or piglets or sus or scrofa or llama or llamas or lama or glama or deer or deers or cervus or elaphus or cow or cows or bos taurus or bos indicus or bovine or bull or bulls or cattle or bison or bisons or sheep or sheeps or ovis aries or ovine or lamb or lambs or mouflon or mouflons or goat or goats or capra or caprine or chamois or rupicapra or leporidae or lagomorpha or lagomorph or rabbit or rabbits or oryctolagus or cuniculus or laprine or hares or lepus or rodentia or rodent or rodents or murinae or mouse or mice or mus or musculus or murine or woodmouse or apodemus or rat or rats or rattus or norvegicus or guinea pig or guinea pigs or cavia or porcellus or hamster or hamsters or mesocricetus or cricetulus or cricetus or gerbil or gerbils or jird or jirds or meriones or unguiculatus or jerboa or jerboas or jaculus or chinchilla or chinchillas or beaver or beavers or castor fiber or castor canadensis or sciuridae or squirrel or squirrels or sciurus or chipmunk or chipmunks or marmot or marmots or marmota or suslik or susliks or spermophilus or cynomys or cottonrat or cottonrats or sigmodon or vole or voles or microtus or myodes or glareolus or primate or primates or prosimian or prosimians or lemur or lemurs or lemuridae or loris or bush baby or bush babies or bushbaby or bushbabies or galago or galagos or anthropoidea or anthropoids or simian or simians or monkey or monkeys or marmoset or marmosets or callithrix or cebuella or tamarin or tamarins or saguinus or leontopithecus or squirrel monkey or squirrel monkeys or saimiri or night monkey or night monkeys or owl monkey or owl monkeys or douroucoulis or aotus or spider monkey or spider monkeys or ateles or baboon or baboons or papio or rhesus monkey or macaque or macaca or mulatta or cynomolgus or fascicularis or green monkey or green monkeys or chlorocebus or vervet or vervets or pygerythrus or hominoidea or ape or apes or hylobatidae or gibbon or gibbons or siamang or siamangs or nomascus or symphalangus or hominidae or orangutan or orangutans or pongo or chimpanzee or chimpanzees or pan troglodytes or bonobo or bonobos or pan paniscus or gorilla or gorillas or troglodytes) 5. #1 AND #2 AND #3 AND #4 |
| China National Knowledge Internet (CNKI)  (N = 139) | TKA=流感+流行性感冒 AND TKA=生物制品+天然药物+传统药物+中医+中药+中草药+草药+植物药+本草+双黄连+热毒宁+银翘散+玉屏风散+冰香散+甘露消毒丹+挥发油+精油 AND TKA=呼吸道投药+呼吸道给药+经鼻+鼻内+鼻用+鼻腔给药+鼻内投药+吹鼻+鼻吸+滴鼻+鼻+吸入+吸入投药+药物的吸入+雾化+雾+喷雾+药物喷雾疗法+吸入装置+吸入器+气雾吸入器+喷雾器+雾化器+雾化器和汽化器+汽化器+芳香+挥发 AND TKA=动物+动物实验+大鼠+小鼠+猪+鼠+羊+兔+猫+狗+牛+鸟+禽+猴+鱼 |
| Wanfang Data Knowledge Service Platform (Wanfang)  (N = 46) | 题名或关键词：("流感"or"流行性感冒") and 题名或关键词：("生物制品"or"天然药物"or"传统药物"or"中医"or"中药"or"中草药"or"草药"or"植物药"or"本草"or"双黄连"or"热毒宁"or"银翘散"or"玉屏风散"or"冰香散"or"甘露消毒丹"or"挥发油"or"精油") and 题名或关键词：("呼吸道投药"or"呼吸道给药"or"经鼻"or"鼻内"or"鼻用"or"鼻腔给药"or"鼻内投药"or"吹鼻"or"鼻吸"or"滴鼻"or"鼻"or"吸入"or"吸入投药"or"药物的吸入"or"雾化"or"雾"or"喷雾"or"药物喷雾疗法"or"吸入装置"or"吸入器"or"气雾吸入器"or"喷雾器"or"雾化器"or"雾化器和汽化器"or"汽化器"or"芳香"or"挥发") and 题名或关键词：("动物"or"动物实验"or"大鼠"or"小鼠"or"猪"or"鼠"or"羊"or"兔"or"猫"or"狗"or"牛"or"鸟"or"禽"or"猴"or"鱼") |
| VIP Information Chinese Periodical Service Platform (VIP)  (N = 135) | (R=呼吸道投药+呼吸道给药+经鼻+鼻内+鼻用+鼻腔给药+鼻内投药+吹鼻+鼻吸+滴鼻+鼻+吸入+吸入投药+药物的吸入+雾化+雾+喷雾+药物喷雾疗法+吸入装置+吸入器+气雾吸入器+喷雾器+雾化器+雾化器和汽化器+汽化器+芳香+挥发) AND (R=生物制品+天然药物+传统药物+中医+中药+中草药+草药+植物药+本草+双黄连+热毒宁+银翘散+玉屏风散+冰香散+甘露消毒丹+挥发油+精油) AND (R=流感+流行性感冒) AND (R=动物+动物实验+大鼠+小鼠+猪+鼠+羊+兔+猫+狗+牛+鸟+禽+猴+鱼) |
| China Biology Medicine Disc (CBM)  (N = 363) | 1. "流感，人"[不加权：扩展] OR "正黏病毒科"[不加权：扩展] OR "流感病毒A型"[不加权：扩展] OR "流感病毒B型"[不加权：扩展] OR "流感"[常用字段：智能] or "流行性感冒"[常用字段：智能] 2. "生物制品"[不加权:扩展] OR "植物, 药用"[不加权:扩展] OR "医学, 传统"[不加权:扩展] OR "挥发油类"[不加权:扩展] OR "生物制品"[常用字段：智能] OR "中医"[常用字段：智能] or "天然药物"[常用字段：智能] or "中药"[常用字段：智能] or "草药"[常用字段：智能] or "植物药"[常用字段：智能] or "传统药物"[常用字段：智能] or "中草药"[常用字段：智能] or "本草"[常用字段：智能] or "双黄连"[常用字段：智能] or "热毒宁"[常用字段：智能] or "银翘散"[常用字段：智能] or "玉屏风散"[常用字段：智能] or "冰香散"[常用字段：智能] or "甘露消毒丹"[常用字段：智能] or "药用植物"[常用字段：智能] or "治疗用植物"[常用字段：智能] or "草药"[常用字段：智能] or "药草"[常用字段：智能] or "挥发油"[常用字段：智能] or "精油"[常用字段：智能] 3. "投药，鼻内"[不加权：扩展] OR "投药，吸入"[不加权：扩展] OR "雾化器和汽化器"[不加权：扩展] OR "芳香疗法"[不加权:扩展] OR "呼吸道投药"[常用字段：智能] OR "呼吸道给药"[常用字段：智能] OR "经鼻"[常用字段：智能] OR "鼻内"[常用字段：智能] OR "鼻用"[常用字段：智能] OR "鼻腔给药"[常用字段：智能] OR "鼻内投药"[常用字段：智能] OR "吹鼻"[常用字段：智能] OR "鼻吸"[常用字段：智能] OR "滴鼻"[常用字段：智能] OR "鼻"[常用字段：智能] OR "吸入"[常用字段：智能] OR "吸入投药"[常用字段：智能] OR "药物的吸入"[常用字段：智能] OR "雾化"[常用字段：智能] OR "雾"[常用字段：智能] OR "喷雾"[常用字段：智能] OR "药物喷雾疗法"[常用字段：智能] OR "吸入装置"[常用字段：智能] OR "吸入器"[常用字段：智能] OR "气雾吸入器"[常用字段：智能] OR "喷雾器"[常用字段：智能] OR "雾化器"[常用字段：智能] OR "雾化器和汽化器"[常用字段：智能] OR "汽化器"[常用字段：智能] OR "芳香"[常用字段：智能] OR "挥发"[常用字段：智能] 4. "动物"[不加权:扩展] or "动物实验"[不加权:扩展] OR "动物"[常用字段：智能] OR "动物实验"[常用字段：智能] OR "大鼠"[常用字段：智能] OR "小鼠"[常用字段：智能] OR "猪"[常用字段：智能] or "鼠"[常用字段：智能] OR "羊"[常用字段：智能] OR "兔"[常用字段：智能] OR "猫"[常用字段：智能] OR "狗"[常用字段：智能] OR "牛"[常用字段：智能] OR "鸟"[常用字段：智能] OR "禽"[常用字段：智能] OR "猴"[常用字段：智能] OR "鱼"[常用字段：智能] 5. #1 AND #2 AND #3 AND #4 |
